# Changes of beneficiaries after Red Cross safe motherhood project in Northern Uganda

**DOI:** 10.4314/ahs.v24i2.24

**Published:** 2024-06

**Authors:** Ai Ogata, Mie Naiki, Yukiya Saito, Alex Onzima

**Affiliations:** 1 Japanese Red Cross College of Nursing, Nursing; 2 Japanese Red Cross Society, International Cooperation Division, Operations Sector; 3 People Empowering People International (PEPI)

**Keywords:** Beneficiaries, Red Cross safe motherhood, Northern Uganda

## Abstract

**Background:**

The Japanese Red Cross Society (JRCS) and the Uganda Red Cross Society (URCS) collaboratively implemented the safe motherhood project for women of reproductive age and community people to improve reproductive health in Northern Uganda from 2010 to 2016. Follow-up visit has not been conducted and the information on detailed changes of the beneficiaries were limited.

**Objectives:**

To explore the beneficiaries' changes after the Red Cross safe motherhood project in Northern Uganda.

**Methods:**

Study design was qualitative content analysis using interview guides.

**Findings:**

After the approval of Institutional Review Board Clearance, the study was started. All participants of the study were informed the study objectives, methods, and ethical considerations and consented to participate. The study participants were six people, three beneficiary couples. Changes in knowledge of safe motherhood and general healthcare, attitude helped and prepared financially, attitude to look women as valuable people, practice of visiting health center to get something, practice of avoiding infectious diseases, practice of increment of ANC visit and facility-based delivery, practice of male involvement for safe motherhood were derived from the interview

**Conclusion:**

Follow-up visit revealed positive changes of beneficiaries after the project, however continuous support is needed for sustainability of the safe motherhood project.

## Introduction

In Acholi (Northern Uganda), over 20 years of internal conflict caused violent assaults and pillage. Therefore, over 2,000,000 internal displaced persons (IDPs), 80% of which were women and children, stayed at IDP camps^1^. In 2009, the maternal mortality ratios were 610 and 550 out of 100,000 births in Acholi and Uganda, respectively^2^. Delivery rates with skilled birth attendants were 16% and 42% in Acholi and Uganda, respectively^2^. Therefore, safe maternal interventions are urgent issues in Acholi. Causes of poor maternal safety include lack of awareness, delay in referring abnormal deliveries, and delay in treatment^3^. The Japanese Red Cross Society (JRCS), responding to requests from the Uganda Red Cross Society (URCS), collaboratively implemented the Safe Motherhood Project for women reproductive age and community people to improve reproductive health in Acholi from 2010 to 2016. The project trained 200 safe motherhood volunteers (Village Health Team) and provided 20,000 Mamabags (safe delivery kits, which included razorblades, cotton, and towels etc.) if they complete antenatal care for four times. The project outcomes were as follows: 1) improved awareness on safe motherhood in the community, and 2) increased antenatal care and delivery rates by skilled birth attendants^3^.

However, follow-up visits have not been conducted, and the information on detailed changes of the beneficiaries were limited. To review the Safe Motherhood Project in northern Uganda, this study aimed to explore the beneficiaries' changes after the project.

## Methods

### Design

This was a descriptive qualitative study.

### Subjects

The Uganda Red Cross Society (URCS) consented to a collaboration with the researcher. Beneficiaries of the Safe Motherhood Project from 2010 to 2016 conducted by the URCS and JRCS in northern Uganda were included. Six participants were selected by purposive sampling, introduced by the URCS Gulu branch, and interviewed one by one.

### Data collection period, study setting, and language

The data collection period was from August 3 to 10, 2017. The study venue was in Gulu (northern area), Republic of Uganda. The spoken languages during the interviews were Acholi (language of northern Uganda) and English. An interpreter translated Acholi statements into English. A qualitative study was conducted through semi-structured interviews with participants using interview guides. Each interview was recorded on an IC recorder.

### Analysis

Verbatim transcripts were created by the transcription company. The data contents were analyzed using data using NVivo 10. Changes and challenges after the Safe Motherhood Project were derived and coded from the beneficiaries' responses. The codes were then sub-grouped and extracted.

### Ethical considerations

The researchers, following the ethical standards of the Declaration of Helsinki, ensured participant and organization autonomy and confidentiality with the approval of the Research Ethics Committee of the Japanese Red Cross College of Nursing (Approval Number: 2017-034). Before the study, the researchers explained the purpose, significance, methods, anonymity, freedom to participate or withdraw, and absence of disadvantages from non-participation. The participants consented to participate and signed a form. The interviews were conducted in the participants' houses where they wanted. The participants were informed that all provided information would only be used for this study. All interviews were recorded with the participants' consent. Recorded data, field notes, and computers used in this study were kept in a locked cabinet in the researcher's office. All personal information were pseudonymized.

The research assistants, who supported the interviews and translated from Acholi to English, were informed about the study ethics and confidentiality by the researchers. Written confidentiality agreement forms were obtained from the assistants. The transcription company also consented and signed the confidentiality agreement at the time of contract between the company and the researchers.

## Results

### Overview of study participants (Table 1)

**Table 1:** Summary of study participants

ID	Affiliation	Education	Gender	Age	Children (*Italic*: Beneficiary Child)
A B	Peasant Farmer	Primary	Husband	30	Boy (7), *Boy (5)*
	Peasant Farmer	Primary	Wife	27	
C D	Peasant Farmer	Primary	Husband	25	Boy (6), *Girl (3)*, Girl (1)
	Peasant Farmer	Primary	Wife	24	
E F	Peasant Farmer	Primary	Husband	35	Girl (11), Girl (10), Girl (8), *Girl (5)*, Girl (7 months)
	Peasant Farmer	Primary	Wife	29	

The study participants were six people, comprising three beneficiary couples. The male and female ages were 25–35 and 24–29 years, respectively. Their occupations were peasant farmers.

According to the study purpose, changes of the beneficiaries after the project were derived and sorted into three categories: knowledge, attitudes, and practice. The following eight sub-categories were derived and extracted as changes after the project, and are presented in Table 2: [Knowledge on safe motherhood], [Knowledge on general healthcare], [Attitude of feeling helped and prepared financially], [Attitude to look women as valuable people], [Practice of visiting health centre to get something], [Practice of avoiding infectious diseases], [Practice of ANC visit and facility-based delivery], and [Practice of male involvement for safe motherhood].

**Table 2:** Changes of the beneficiaries after the Safe Motherhood Project in Northern Uganda

Category	Sub-category	Code
Knowledge	Safe motherhood	Look at ladies like valuable people
		Helping woman and male involvement during pregnancy
		Family planning
		Importance of 4-times ANC visit and facility-based delivery
		Breast feeding and child nutrition
		HIV/AIDs testing
		Sleeping under mosquito nets
	General health care	Childcare
		Hygiene
Attitude	Feeling helped and prepared financially	No money to buy cotton, clothes for baby
		Afford to prepare necessary items for delivery
	Look women as valuable people	Share ideas and plans with spouse
		Woman as valuable people
Practice	Visiting health center to get something	Mamabag as a morale booster
		People who saw mothers carrying Mamabags began to visit the health center
		There is something good they are going to get in HC.
	Avoiding infectious diseases	Screening at HC
		Preventive actions to sexual transmitted diseases, malaria, and other infectious diseases
	ANC visit and facility-based delivery	Complete ANC for 4 times
		Deliver at health facilities
	Male involvement for safe motherhood	Husbands visit health center for ANC, HIV test, and delivery escorting wives
		Child spacing
		No gender-based violence toward women
		No polygamy
		Share housework
		Share ideas among couples

### Change [Knowledge on safe motherhood]

Acquiring knowledge on safe motherhood was the change after the project. Beneficiaries were taught about actions on helping women, male involvement during pregnancy, family planning, importance of ANC visit, facility-based/skilled personnel-attended delivery, breastfeeding, and HIV-AIDS testing.

*“The program encouraged mothers so much to go to the health centre for antenatal and we learned about child's bathing during that time”* (A)

*“The most impressive input was the information, the dialogue, we had about putting an end to domestic violence. That's why I am very happy in my house right now together with my husband”* (B)

*“It created awareness. Child spacing. It also created awareness on how we can take care of our children. It strengthened antenatal care, men involvement in antenatal care. Those days, I was not knowledgeable about antenatal [care]. I felt [that] antenatal [care] was not very important, but when this program came in, it encouraged me to go.”* (C)

### Change [Knowledge on general healthcare]

Apart from the specific knowledge on safe motherhood, beneficiaries were taught about general healthcare knowledge on hygiene and childcare.

*“I learned about hygiene. I was taught the importance of having a big latrine, taking good care of the compound”* (F)

### Change [Attitude of feeling helped and prepared financially]

Developing an attitude of feeling financially helped and prepared was one effect of the project. Before the project, beneficiaries suffered from poverty after the conflict and could not afford necessary items for delivery. Moreover, people did not have enough financial resources for events such as childbirth.

*“What motivated me so much is the Mamabag because during that time, we never had enough money to shop all these materials to take care of the baby, so when we were given soap[,] it really supported us so well and it really motivated me to go in together with my wife so that we get….”* (E)

### Change [Attitude to look women as valuable people]

Project changed the beneficiaries' attitude to look women as a valuable people although men had used to neglect women.

*“When that program was not yet introduced, men used to neglect wives. But when they brought that program[,] at least it [boosted the morale of the] men and right now they could escort their women for antenatal. Sometimes when you go to the health center alone without your husband, they don't test you. So men were encouraged during that program to escort their women. Men learnt the concept of helping their woman during pregnancy, delivery and breastfeeding”* (C)

### Change [Practice of visiting the health centre to get something]

<Mamabag as a morale booster>

Mamabag itself promoted safe motherhood because the contents were very attractive for beneficiaries.

*“Mamabag could [boost our morale]. We feel loved when we are given [contents] from the health centre.”* (D)

<Benefits received from the health centre>

Small incentives attracted beneficiaries to visit the health centre.

*“It encouraged all women to go to the health centre because we know whenever we reach there, there is something good we are going to get”* (D)

### Change [Practice of avoiding infectious diseases]

After the project, the participants' preventive behaviour and early consultation practices were promoted.

<Screening at the health centre>

*“Previously, I was so scared [of proceeding with my wife] for HIV testing and counselling, but for the first time when I tested and found I was negative, it really encouraged me so much”* (A)

<Preventive actions for sexually transmitted diseases, malaria, and other infectious diseases>

*“We started sleeping [with] treated mosquito nets”* (F)

*“Up to now, we are not yet infected with any infections that people get during intercourse or any other thing”* (E)

### Change [Practice of ANC visit and facility-based delivery]

The project increased the ANC coverage and facility-based delivery.

*“With the first child, I attended antenatal only once and I then delivered from home. The second child, I was having – I was most of the time sick so I kept going to the hospital [for antenatal consults] five times. With the third child, I only [checked up thrice] because I started very late, yes, and my husband used not to escort me. [For] the fourth child, I went for four times together with my husband with the help of the Village Health Team's advice”* (F) the families to change their practices, space their children, and take good care of them.

**Figure 1:**
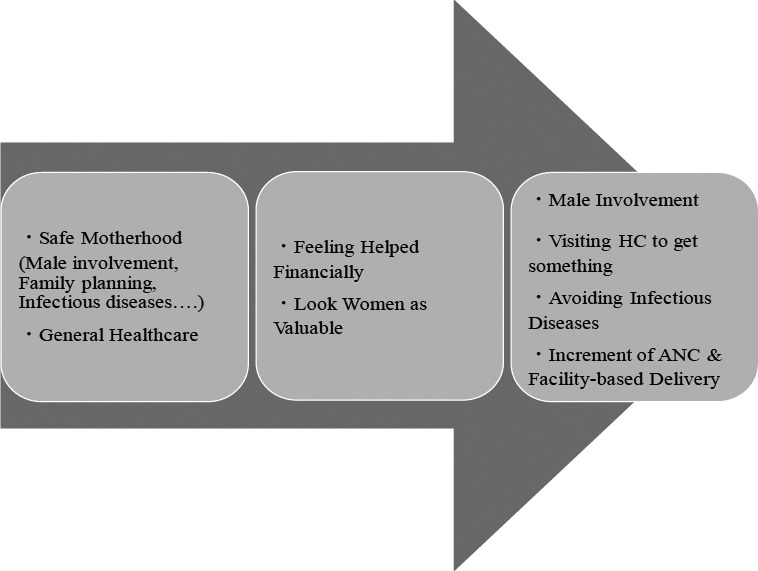
Changes of the beneficiaries' knowledge, attitudes, practice after the safe motherhood project in northern Uganda

Within six years since the start of the project, knowledge has become entrenched in people and communities, and has been transformed into practice (Figure 1). To continue these practices, it is necessary to continue long-term support over longer time periods.

There are other vital issues aside from a longer term of support, such as developing reproductive health professionals, improving access to health centres, and resolving poverty. Based on this study results, the researchers suggest a program for reproductive health professionals. Improving access is inevitable for both beneficiaries and health care professionals. By adding health centre staff, regular home visits would increase, without waiting for beneficiaries to come to the health centre. Resolving poverty will increase health centre check-ups and prevent delivery-related complications.

As the strategic plan of the Red Cross/Crescent and the Sustainable Development Goals (SDGs) focus on reproductive health in Africa, the researchers realized that it was important to discuss the program of reproductive health and contribute to this area.

### The significance of the study is as follows

#### Sustainability development by monitoring

This study was a collaborative study with the URCS, URCS volunteers, and beneficiaries in the community in northern Uganda. The researchers believe that this study would sensitize and motivate the participants to continue their safe motherhood activities.

#### For future project possibilities for future mothers and fathers

The Safe Motherhood Project in northern Uganda benefitted not only the women in their reproductive age but also their children. The researchers suggest possibilities for the study to include future mothers and fathers.

#### Contribution to SDGs 3 and 5

This study will contribute to the sustainability of safe motherhood in Uganda, attaining SDGs 3 (good health and well-being) and 5 (gender equality) in African countries.

## Conclusion

The project succeeded in changing the beneficiaries' knowledge, attitudes, and behaviours. Beneficiaries came to the health centre and promoted male involement using incentives as rewards for visiting the health centre. Incentives played a big role to motivate the beneficiaries; however, the sustainability of these preferrable practices even without incentives would be a challenge.

## References

[1] Lord's Resistance Army Disarmament and Northern Uganda Recovery Act of 2009 (2010).

[2] United Nations Children's Fund, Uganda Country Profile (2009).

[3] Ogata A, Onzima A, Saito U, Ueda M (2014). Report of Collaborative Safe Motherhood Project (Japanese Red Cross and Uganda Red Cross Society) phase 1 in northern Uganda. The 39^th^ Annual Meeting (Western Japan Branch). Japan Association for International Health.

[4] Hasahya OT, Berggren V, Sematimba D, Nabirye RC, Kumakech E (2016). Beliefs, perceptions and health-seeking behaviours in relation to cervical cancer: a qualitative study among women in Uganda following completion of an HPV vaccination campaign. Global Health Action.

